# Immunohistochemical expression of Ki-67, INSM1, and SSTR2A in medullary thyroid carcinoma: correlation with tumour size, vascular invasion, and biochemical outcome

**DOI:** 10.1007/s00428-025-04287-z

**Published:** 2025-10-25

**Authors:** Esther Diana Rossi, Salvatore Raia, Mariagrazia Maratta, Natalia Cappoli, Antonino Mulé, Pierfrancesco Oliva, Giovanni Aulino, Sabrina Chiloiro, Domenico Milardi, Ernesto Rossi, Francesco Pennestrì, Marco Raffaelli, Giampaolo Tortora, Alfredo Pontecorvi, Antonio Bianchi, Giovanni Schinzari

**Affiliations:** 1 Division of Anatomic Pathology and Histology Fondazione,, Policlinico Universitario “Agostino Gemelli”-IRCCS, Rome, Italy; 2 Internal Medicine, Endocrinology and Metabolism Unit, Fondazione Policlinico Universitario Agostino Gemelli-IRCCS, Department of Translational Medicine and Surgery, Università Cattolica del Sacro Cuore, Rome, 00168 Italy; 3 Medical Oncology Unit, Fondazione Policlinico Universitario “A. Gemelli” IRCCS, Department of Translational Medicine and Surgery, Rome, Italy; 4 Department of Health Care Surveillance and Bioethics, Section of Legal Medicine, Università Cattolica del Sacro Cuore, Rome, Italy; 5 Metabolic and Endocrine Surgery Unit, Fondazione Policlinico Universitario Agostino Gemelli, IRCCS, Department of Translational Medicine and Surgery, Università Cattolica del Sacro Cuore, Rome, 00168 Italy

**Keywords:** Medullary thyroid carcinoma (MTC), Ki-67, INSM1, SSTR2A, Immunohistochemistry, Prognosis

## Abstract

In the management of medullary thyroid carcinoma (MTC), current prognostic tools have limited discriminatory power, and no widely accepted tissue markers are available aside from proliferative activity and tumour necrosis, recently validated in a grading system. Herein, we assess the prognostic value of selected immunohistochemical biomarkers in MTC: proliferation index Ki-67, insulinoma-associated protein 1 (INSM1), and somatostatin receptor subtype 2A (SSTR2A). We retrospectively analysed 43 patients diagnosed at our centre between October 2003 and July 2024 with histologically confirmed MTC (mean follow-up, 52.5 months). Expression of Ki-67 (> 3%), INSM1, and SSTR2A was evaluated on formalin-fixed, paraffin-embedded tissue sections. Associations between these biomarkers and clinicopathological variables, as well as biochemical cure, were analyzed. Additionally, we compared 13 patients with double tumours (DT)—with medullary and papillary/follicular components—to the remaining 30 patients to identify significant differences. A Ki-67 index > 3% significantly correlated with larger tumour size (26.0 mm vs 9.6 mm; *p* < 0.001) and vascular invasion (80% vs 31.6%; *p* = 0.035). INSM1 expression was associated with larger tumour size (12.9 mm vs 3.2 mm; *p* = 0.021), vascular invasion (43.2% vs 0%; *p* = 0.042), and lower biochemical cure rate (54.1% vs 100%; *p* = 0.033). SSTR2A expression correlated with biochemical cure (100% vs 54.1%; *p* = 0.033) and was more frequent in DT patients (66.7% vs 24.3%; *p* = 0.036). Among all comparisons, only SSTR2A expression significantly differed between DTs and other tumours. In conclusion, Ki-67 and INSM1 identify patients with more aggressive MTC, while SSTR2A defines a favourable subgroup with complete remission.

**Supplementary Information:**

The online version contains supplementary material available at 10.1007/s00428-025-04287-z.

## Introduction

Medullary thyroid carcinoma (MTC) is a rare neuro-endocrine malignancy that arises from para-follicular C cells and represents roughly 4–5% of all thyroid cancers worldwide [1]. Although total thyroidectomy with prophylactic central-neck dissection can be curative, postoperative courses differ strikingly: some patients experience decades-long biochemical remission, whereas others progress within a few years. The 8th-edition AJCC/TNM system remains the benchmark for initial risk stratification, yet external validations show that stage groups overlap considerably in long-term survival [2]. Consequently, clinicians still struggle to identify—at the time of surgery—the minority of patients destined for aggressive disease. On the biochemical side, serial doubling times of serum calcitonin and carcino-embryonic antigen (CEA) are reliable surrogates of tumour kinetics, but they require months of follow-up and therefore cannot influence immediate postoperative decisions [3]. A recent umbrella review, according to last ATA guidelines suggestions (4–5), report that a basal calcitonin ≥ 10 pg/ml may be an accurate single threshold to detect residual disease, yet even this cut-off lacks granularity in forecasting future progression [4, 5]. Attention has therefore shifted to tissue-based markers that can be scored directly on the surgical specimen and be useful to predict the clinical history of the disease. Ki-67 is a widely used proliferation marker in endocrine pathology. Fuchs et al. analysed 140 MTCs and demonstrated that Ki-67 3 % cut-off segregated tumours with higher mitotic counts, necrosis, and inferior survival [6]. Building on that work, the *International Medullary Thyroid Carcinoma Grading System (IMTCGS)* now incorporates Ki-67 alongside mitotic rate and necrosis; a threshold of *5%* separates low- from intermediate-grade lesions, while ≥ 20% defines high-grade disease [7]. The IMTCGS was validated across four international cohorts and retained prognostic power irrespective of stage or RET status, underscoring Ki-67 as a core biological variable rather than a mere epiphenomenon of tumour burden. Despite this progress, the practical impact of low-range Ki-67 values (3–5%), which are common in routine pathology, has not been fully clarified. *Somatostatin receptor-2A (SSTR2A)* is expressed in approximately one-third of sporadic MTCs; in the largest immunohistochemical series to date, de Vries et al. showed that membranous SSTR2A positivity correlated with longer overall survival, even after adjustment for stage [8]. *Insulinoma-associated protein-1 (INSM1)* is a transcription factor essential for neuro-endocrine differentiation. Its crisp nuclear staining pattern yields > 90 % sensitivity and near-absolute specificity for MTC, outperforming traditional cytoplasmic markers such as chromogranin and synaptophysin [9]. Diagnostic value notwithstanding, little is known about its prognostic significance. INSM1 over-expression has been linked to poor outcome in nasopharyngeal carcinoma, suggesting that it may capture a transcriptional programme of aggressive growth [10]. A less investigated aspect of MTC biology is the rare coexistence of medullary and other thyroid carcinoma components within the same tumour, with reported prevalences generally below 3%, and only occasionally reaching 7% in single-centre series [11].Two, not mutually exclusive, models are debated: (a) *collision tumours* arising from independent clonal populations, and (b) divergence from a *bipotent endodermal progenitor*, a concept supported by lineage-tracing experiments in mice that relocate C-cell ontogeny from the neural crest to the anterior endoderm [12]. Over the past three decades, the incidence of MTC has risen slightly and surgical management has become more radical, translating into modest gains in disease-specific survival [13]. The *2022 WHO classification* now formalises a two-tier grading scheme that integrates mitotic rate, necrosis, and Ki-67 index [14]. Clinical practice is still guided by the 2015 American Thyroid Association (ATA) recommendations [5], but several updates have refined peri-operative management and long-term surveillance strategies [15, 16]. On the molecular front, long-term screening has clarified *RET* genotype–phenotype correlations in hereditary disease [17, 18], whereas next-generation sequencing of sporadic tumours reveals mutually exclusive *RET* or *RAS* driver mutations and activation of the PI3K–Akt–mTOR pathway [19–21]. These insights underpin an expanding systemic arsenal: *vandetanib* and *cabozantinib* were the first multitarget kinase inhibitors to improve progression-free survival in advanced MTC [22, 23]; more recently, the selective RET inhibitors *selpercatinib* and *pralsetinib* have produced durable responses in RET-altered disease with a favourable safety profile [24, 25]. Despite these advances, clinicians still lack a simple, specimen-based tool that distinguishes indolent from high-risk MTC immediately after surgery. Reviews of molecular prognostic markers highlight opportunities to augment TNM staging, yet consensus on how to incorporate these variables into routine care is missing [26]. We therefore investigated whether a three-marker panel—Ki-67 (cut-off: 3%), insulinoma-associated protein 1 (INSM1), and somatostatin receptor subtype 2 A (SSTR2A) —may improve postoperative risk stratification in medullary thyroid carcinoma (MTC) by correlating their expression with histopathological features and clinical prognosis.

## Materials and methods

### Study design and structure

This retrospective single-centre cohort study was conducted at Policlinico Universitario Agostino Gemelli IRCCS, Rome. A total of 43 consecutively enrolled patients histologically diagnosed with medullary thyroid carcinoma (MTC) between October 2003 and July 2024 were analysed, drawing clinical, pathological, and biomarker-related data from electronic medical records, surgical reports, pathology reports, and follow-up visits. All the cases were histologically revised, and we chose a single sample for the performance of immunomarkers which were specifically done for this study. The same was done for the KI-67 analysis. The cohort was observed for a mean follow-up of 52.5 months. Data extraction and analysis for this work were carried out between February 2025 and May 2025. All information was systematically collected from clinical records at Policlinico Universitario Agostino Gemelli, ensuring a comprehensive evaluation of clinical, pathological, and biomarker data to investigate correlations with patient outcomes.

Variables collected included:

- Anthropometric and demographic variables: age, sex
- Clinical variables: stage at diagnosis, type of carcinoma (pure medullary vs “double tumours” which means two distinct tumours coexist within the same thyroid gland: MTC and non-medullary differentiation, such as papillary or follicular thyroid carcinoma).
- Surgical and pathological variables: tumour size, extrathyroidal extension, vascular invasion, perineural invasion, encapsulation, capsular infiltration, surgical margins, multifocality, desmoplasia.
- Biomarkers: Ki-67 expression (%), INSM1 expression (positive/negative), SSTR2A expression (positive/negative). All these markers were specifically stained and evaluated for this study.
- Imaging variables: fluorodeoxyglucose-positron emission tomography (FDG-PET), gallium-68 positron emission tomography (Gallium-PET), and 6-[18F] fluoro-L-3,4-dihydroxyphenylalanine positron emission tomography (DOPA-PET) scan results.
- Genetic variables: somatic RET mutations, MEN2A/MEN2B syndrome status
- Biochemical variables: calcitonin and carcinoembryonic antigen (CEA) levels pre- and post-surgery and at last follow-up
- Therapeutic variables: systemic therapies
- Follow-up and outcome variables: DFS, PFS, follow-up duration, final status (cured/not cured)
- Comorbidities: hypertension, diabetes, osteoporosis, dyslipidemia, pheochromocytoma, hyperparathyroidism, other non-thyroid neoplasms

Inclusion criteria:

- Confirmed histopathological diagnosis of medullary thyroid carcinoma
- Availability of comprehensive clinical, surgical, pathological, biomarker, and follow-up data
- Age greater than 18 years at the time of diagnosis

Exclusion criteria:

- Incomplete clinical, surgical, or pathological records
- Lack of essential biomarker data
- Age younger than 18 years at diagnosis

Patients not meeting these criteria were excluded to ensure homogeneity and reliability of the results.Patients not meeting these criteria were excluded to ensure homogeneity and reliability of the results.

### Histology

For the surgical samples, we use the standard fixation in 10% buffered formaldehyde, followed by paraffin and then subsequent 5-µm-thick sections stained with haematoxylin and eosin (H&E). In all the cases, we had peri-thyroid adipose tissue that was entirely examined for searching lymph nodes. The histological criteria adopted for the diagnosis of MTC and other tumours were based on the 5th edition of the WHO 2022 [14]. The eighth edition of the tumour-node-metastasis-based staging system recommended by the American Joint Commission on Cancer (AJCC) was used in all the cases [26].

*Ki-67*: According to the 5th ed of the WHO, the cases were classified into low-grade and high-grade MTC according to (a) Ki-67 ≥ 5%, (b) > 5 mitotic figures per 2 mm^2^, and (c) necrotic component. However, based on the evidence that many cases had a Ki-67 between 0 and 5% (< 5%), we decided to also adopt an intermediate value of 3% and a further classification of Ki-67 was evaluated and compared with the standard 5%. None of the cases fell into the gap between 3 and 5%. For this reason, all the tumours were further dichotomised using a *3% Ki-67 cut-off*.

*Biochemical persistence* was defined as a calcitonin level ≥ *10 pg/ml*. This threshold maximises sensitivity for residual disease while limiting false-positive results.

*Double tumours were defined as* two distinct malignant neoplasms coexisting within the same thyroid gland: one of medullary origin and the other of non-medullary differentiation, such as papillary or follicular thyroid carcinoma.

### Laboratory methods

Data utilised in this study were collected from electronic medical records available at Policlinico Universitario Agostino Gemelli.

Laboratory and pathological assessments included: Immunohistochemistry (IHC) for the biomarkers Ki-67 (clone 30–9, pre-dilute RRID: 05278384001; Roche Diagnostic SPA), INSM1 (INSM1 clone A-8, dilution 1:50, RRID: MOB568-05; GennovaScientific), and SSTR2A (clone UMB1, dilution 1:500, RRID: AB 134152; Abcam), which were performed on formalin-fixed paraffin-embedded (FFPE) tumour tissues. In brief, 3–4-μm-thick sections were obtained from paraffin-embedded tissue blocks and immunostained by using the automated platforms Agilent Dako Omnis and Roche VENTANA® Benchmark Ultra, through an automated procedure. Ki-67 was quantified as the percentage of tumour cells showing positive staining. INSM1 and SSTR2A expression were evaluated qualitatively (positive/negative), with SSTR2A assessed based on membranous immunoreactivity of tumour cells. Negativity was defined by the lack of positivity for the immunomarkers in all the neoplastic cells. They were performed on one single selected slide of MTC. The “INSM1 negative internal control” refers to a control sample, such as foetal liver tissue or normal mesenchymal tissue, used in experiments to confirm that no INSM1 protein is present or detected when it is expected to be absent. On the other hand, SSTR2 expression in pancreatic islet cells and pancreatic acinar cells from the same tissue block can serve as a negative control for SSTR2 expression, ensuring that the positive signal from islet cells is real. Genetic testing for somatic RET mutations and screening for MEN2A and MEN2B syndromes were performed according to institutional protocols. Serial biochemical measurements of serum calcitonin and carcinoembryonic antigen (CEA) levels were used as both a diagnostic tool and for follow-up monitoring. Follow-up evaluations included regular imaging studies (ultrasound, computed tomography, positron emission tomography) and biochemical assessments to monitor disease status. Disease-free survival (DFS) and progression-free survival (PFS) were calculated from the date of surgical intervention or initiation of systemic therapy to the date of disease recurrence or progression, respectively. Clinical outcomes were determined based on the presence or absence of structural and/or biochemical evidence of disease at the last follow-up.

### Statistical analysis

All analyses were performed using STATA software (version 18, StataCorp, College Station, TX, USA). A descriptive analysis of the study population was first conducted.

Categorical variables were expressed as absolute frequencies (*n*) and percentages (%), and continuous variables were summarised as mean, standard deviation, variance, skewness, and kurtosis.

Associations between clinicopathological features and the expression of the biomarkers Ki-67, INSM1, and SSTR2A were initially assessed in univariate analyses. For categorical variables, Pearson’s chi-square test was used; when variables included more than two categories, the global Pearson’s test was applied. For continuous variables, univariate comparisons were performed using linear regression models.

Comparisons between patients with double tumours (coexisting MTC and non-medullary thyroid carcinoma in the same gland) and those with isolated MTC were carried out using the same univariate framework.

Variables with *p* < 0.10 in univariate analyses were subsequently entered into multivariable logistic regression models, adjusting for relevant clinicopathological covariates. Multicollinearity was assessed using variance inflation factors (VIF). The relationship between disease outcome (cured vs not cured) and the presence of comorbidities was examined using logistic regression models (univariate and multivariable). All regression models report odds ratios (OR), 95% confidence intervals (CI), and *p*-values. To account for multiple comparisons, *p*-values were corrected using the Bonferroni method. Statistical significance was defined as a two-sided corrected *p*-value < 0.05.

### Ethics

This study was conducted in accordance with the Declaration of Helsinki and approved by the Ethics Committee of CET Lazio Area 3 (protocol ID 6506 approved on 31 October 2024).

## Results

### Patients’ characteristics (supplementary materials Tables S1–S4)

**Fig. 1:**
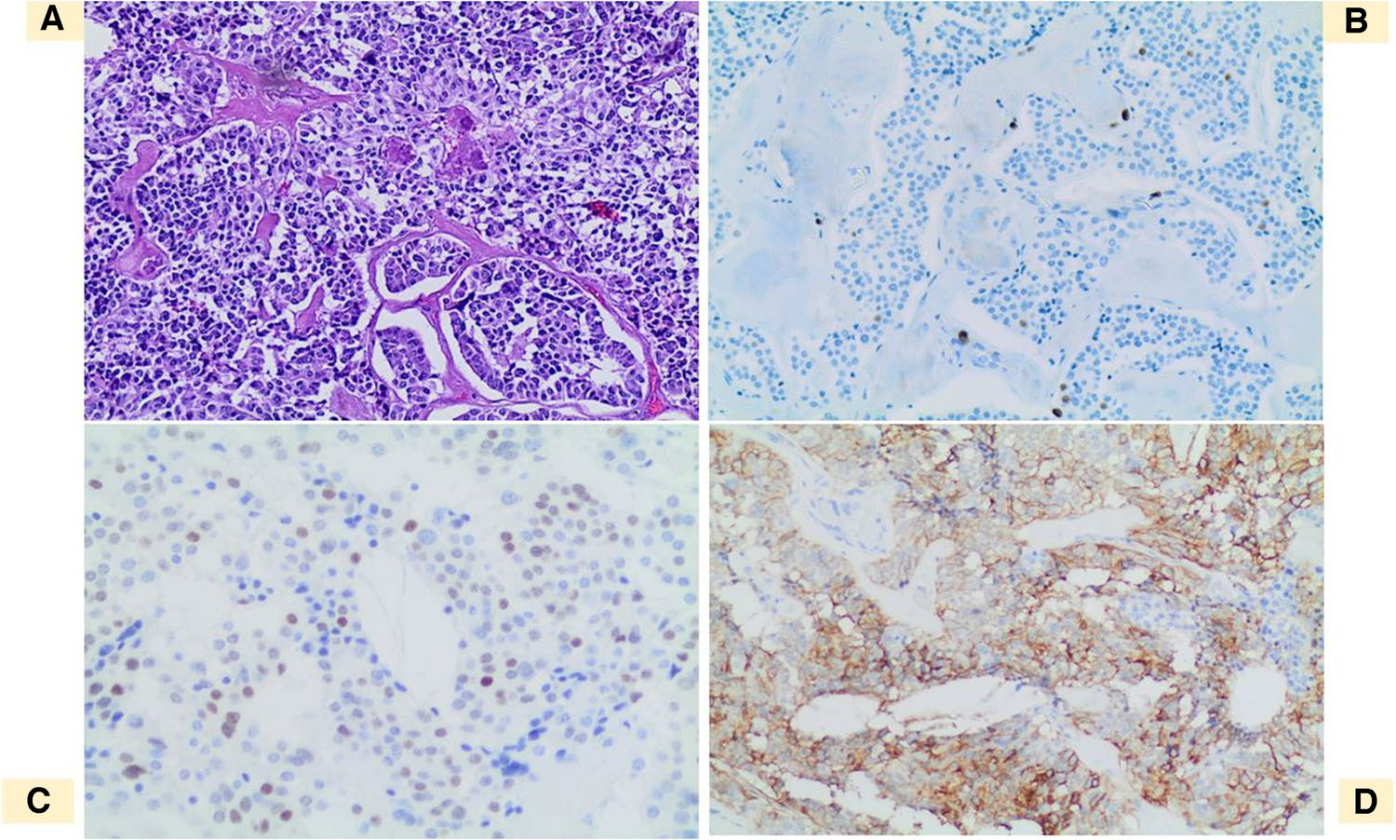
**A**–**D** **A** The morphological (architectural and cellular) details of an MTC (H&E ×200). **B** The Ki-67 < 3% (AB ×200). **C** The nuclear expression of INSM1 which was labeled as 95% in this case (AB, ×200). **D** The membranous-cytoplasmic expression of SSTR2A, which was defined as 70–75% in this case (AB, ×200)

**Fig. 2:**
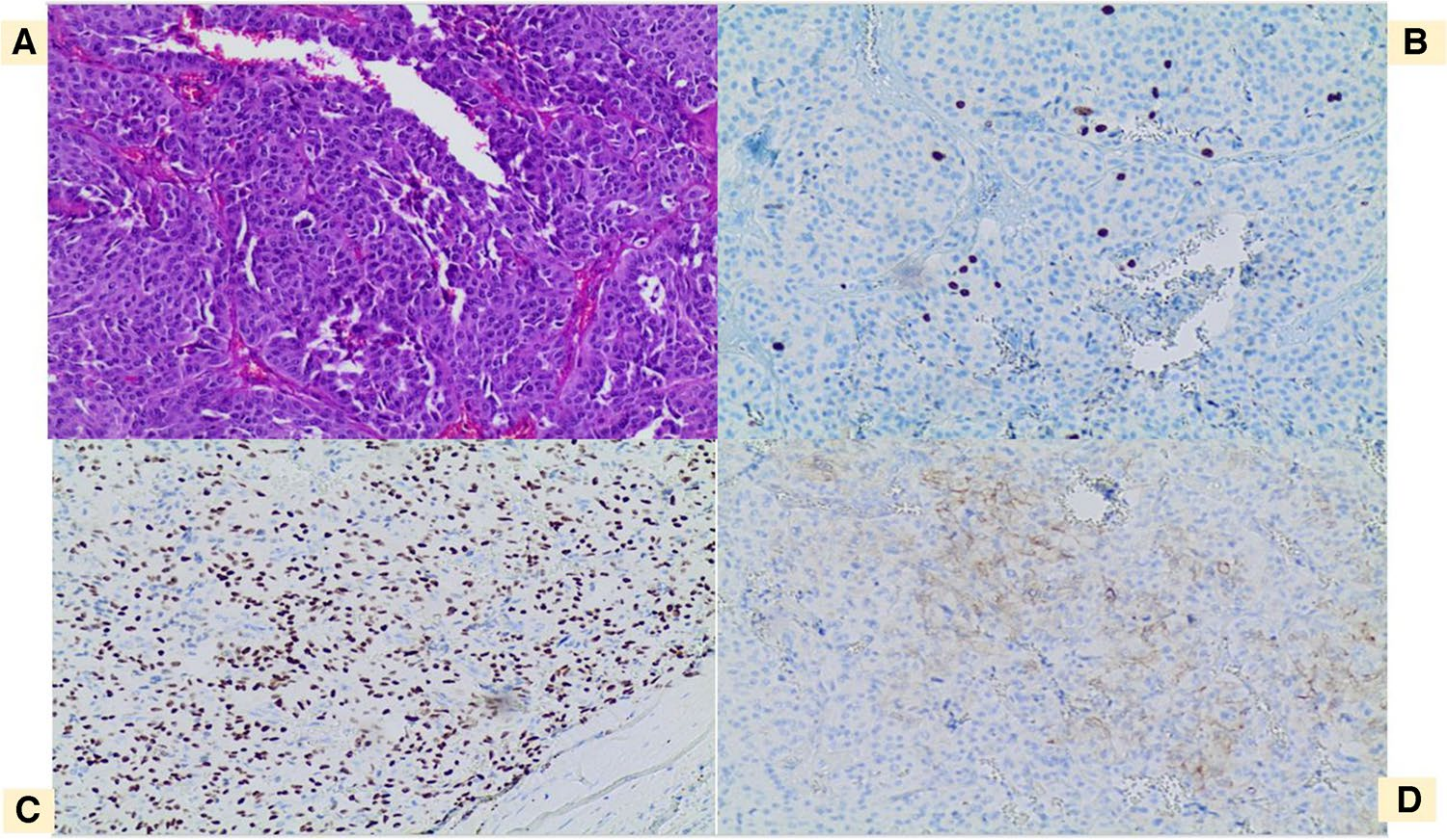
**A**–**D** **A** The morphological (architectural and cellular) details of an MTC (H&E ×200). **B** The Ki-67 5% (AB ×200). **C** The nuclear expression of INSM1 which was labeled as 100% in this case (AB, ×200). **D** The membranous-cytoplasmic expression of SSTR2A, which was defined as focal 2–5% in this case (AB, ×200)

**Fig. 3:**
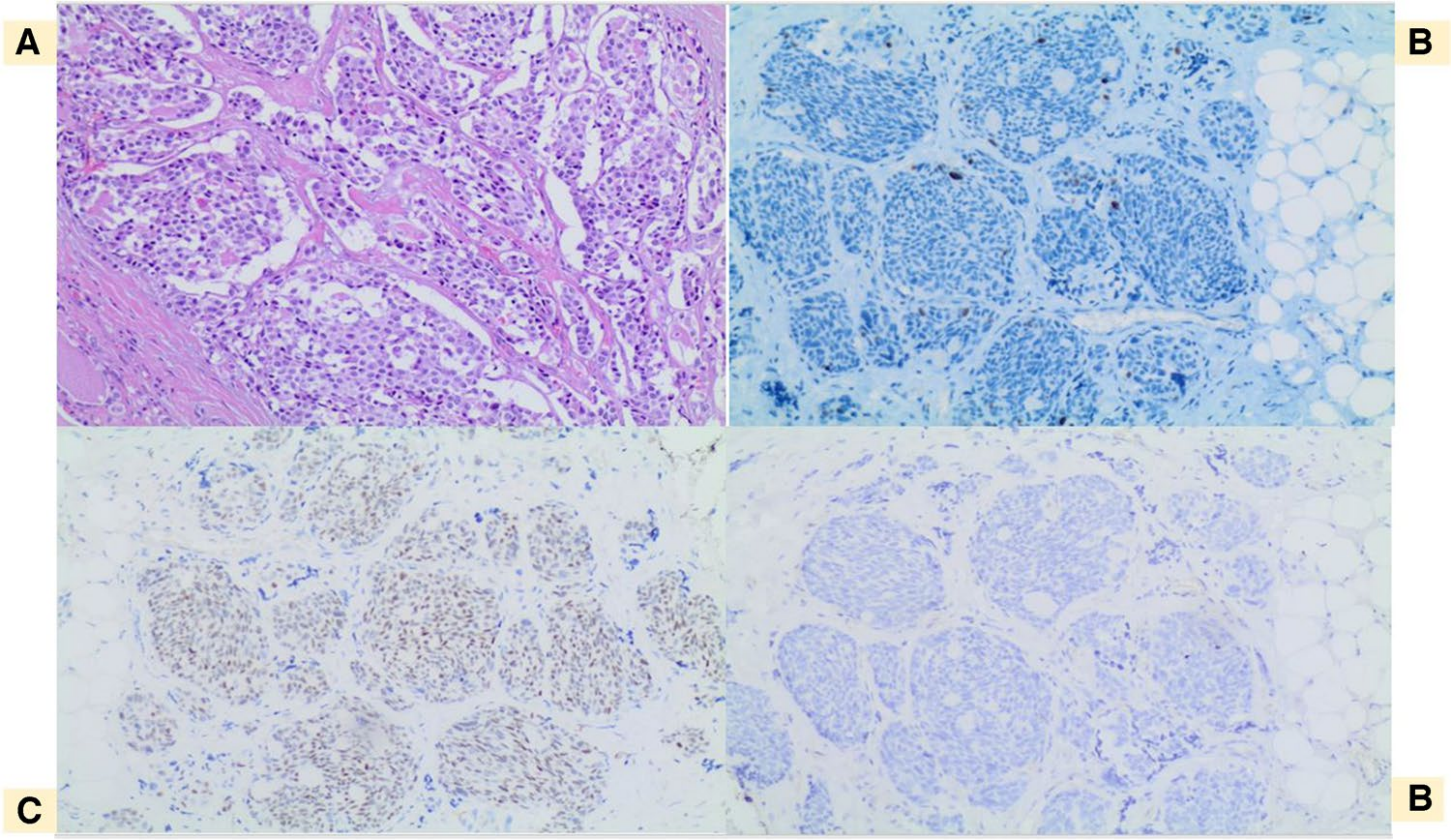
**A**–**D** **A** The morphological (architectural and cellular) details of an MTC (H&E ×200). **B** The Ki-67 < 1% (AB ×200). **C** The nuclear expression of INSM1 which was labeled as 100% in this case (AB, ×200). **D** Negative SSTR2A (AB, ×200)

We retrospectively analysed 43 patients with a histological diagnosis of MTC who were treated at our centre between October 2003 and July 2024. Baseline clinicopathological data are summarised in Supplementary Materials Tables S1–S4. The cohort comprised 26 women (60.5%) and 17 men (39.5%) with a median age at diagnosis of 54 years (IQR 42–66). Mean primary-tumour size was 11.5 ± 9.6 mm. TNM stage at diagnosis was I in 26 patients (60.5%) and IVA–IVC in 12 (27.9%); distant metastasis was present in 2 (4.7%). Double tumours occurred in 13/43 cases (30.2%). Extrathyroidal extension, vascular invasion, perineural invasion, and desmoplasia were identified in 3/43 (7.0%), 16/43 (37.2%), 2/43 (4.7%), and 3/43 (7.0%) tumours, respectively. The most common comorbidities were hypertension (35.7%) and dyslipidaemia (32.6%)**.** Overall biochemical cure rate was 26/43 (60.5%). Mean DFS for the entire cohort was 49.1 ± 62.9 months; PFS (analysed in three patients with persistent disease) was 48.7 ± 66.2 months. The mean follow-up was 52.5 months during which other continuous variables such as calcitonin and CEA levels were recorded (Figs. 1, 2 and 3).

### Biomarker expression

Ki-67 > 3% in 5/43 tumours (11.6%), INSM1 positive in 37/43 (86.0%), SSTR2A positive in 6/43 (14.0%).

Ki-67 correlations (Table 1, Supplementary Materials Figures SA and SB).

**Table 1:** Ki-67 proliferation index

	Ki-67 0–3%	Ki-67 > 3%		Total	Test (*p*-value)	Odds ratio (OR) 95% CI
Type Only medullary cancer, *N* (%) Double tumours *N* (%)	26 (68.4%) 12 (31.6%)	4 (80.0%) 1 (20.0%)		30 (69.8%) 13 (30.2%)	0.596	
Primary tumour size (mm) Mean (SD)	9.611 (5.829)	26.000 (19.026)		11.516 (9.626)	< 0.001	1.128738 1.001114—1.272631
Extrathyroid extension No, *N* (%) Yes, *N* (%)	36 94.7%) 2 (5.3%)	4 (80.0%) 1 (20.0%)		40 (93.0%) 3 (7.0%)	0.224	
Vascular invasion No, *N* (%) Yes,* N* (%)	26(68.4%) 12 31.6%)	1 (20.0%) 4 (80.0%)		27 (62.8%) 16 (37.2%)	0.035	2.949622 0.2222563—39.14522
Perineural invasion No, *N* (%) Yes, *N* (%)	37 97.4%) 1 (2.6%)	4 (80.0%) 1 (20.0%)		41 (95.3%) 2 (4.7%)	0.083	
Encapsulated neoplasm No, *N* (%) Yes, *N* (%)	37 97.4%) 1 (2.6%)	4 (80.0%) 1 (20.0%)		41 (95.3%) 2 (4.7%)	0.083	
Capsular infiltration No, *N* (%) Yes, *N* (%)	37 97.4%) 1 (2.6%)	4 (80.0%) 1 (20.0%)		41 (95.3%) 2 (4.7%)	0.083	
Surgical margins Negative, *N* (%) Positive,* N* (%)	37 97.4%) 1 (2.6%)	4 (80.0%) 1 (20.0%)		41 (95.3%) 2 (4.7%)	0.083	
Multifocality No, *N* (%) Yes, *N* (%)	20 52.6%) 18 47.4%)	2 (40.0%) 3 (60.0%)		22 (51.2%) 21 (48.8%)	0.595	
Stage at diagnosis, *N* (%) I II III IVa IVb IVc	24 63.2%) 1 (2.6%) 3 (7.9%) 8 (21.1%) 0 (0.0%) 2 (5.3%)	2 (40.0%) 1 (20.0%) 0 (0.0%) 2 (40.0%) 0 (0.0%) 0 (0.0%)	26 (60.5%) 2 (4.7%) 3 (7.0%) 10 (23.3%) 0 (0.0%) 2 (4.7%)		0.331	
DFS (months) Mean (SD)	49.914 (65.761)	43.400 (42.241)	49.100 (62.912)		0.832	
PFS (months) Mean (SD)	48.667 (66.154)	0.0 (0.0)	48.667 (66.154)			
Pre-surgery calcitonin, pg/ml Mean (SD)	1571.726 (6799.057)	2995.600 (4270.062)	1737.293 (6532.515)		0.652	
Post-surgery calcitonin, pg/ml Mean (SD)	142.686 (525.703)	742.400 (1570.360)	212.420 (718.444)		0.079	
Last follow-up calcitonin, pg/ml Mean (SD)	181.317 (640.564)	215.400 (286.512)	185.280 (607.795)		0.908	
Last follow-up CEA, ng/ml Mean (SD)	18.753 (67.915)	179.884 (365.516)	37.489 (139.710)		**0.013**	
Last follow-up status, *N* (%) Cured Not cured	24(63.2%) 14(36.8%)	2 (40.0%) 3 (60.0%)	26 (60.5%) 17 (39.5%)		0.319	

Basing on the WHO 2022 classification cut-off (Ki-67 ≥ 5%) we identified only 2/43 tumours (4.7%) as high grade, with no significant clinicopathological or survival statistical differences in our cohort. To further improve stratification, we then adopted a Ki-67 > 3% cut-off and after statistical analysis we found it was associated with:

- Larger tumour size: 26.0 ± 19.0 mm vs 9.6 ± 5.8 mm (*p* < 0.001).
- More frequent vascular invasion: 80.0% vs 31.6% (*p* = 0.035; OR 2.95, 95% CI 0.22–39.15).

No significant associations were observed for double tumours, extrathyroid extension, perineural invasion, capsular infiltration, surgical-margin status, multifocality, AJCC stage, DFS or PFS, and outcome (all *p* > 0.05), as well as desmoplasia in MTC and the genetic profile of the tumours.

INSM1 correlations (Table 2, Supplementary Materials Figures SE, SF, and SG).

**Table 2:** NSM1

	INSM1 negative	INSM1 positive	Total		Test (*p*-value)
Number of patients, *N* (%)	6 (14.0%)	37 (86.0%)	43 (100.0%)		
Type Only medullary cancer, *N* (%) Double tumours *N* (%)	5 (83.3%) 1 (16.7%)	25 (67.6%) 12 (32.4%)	30 (69.8%) 13 (30.2%)		0.435
Primary tumour size, mm Mean (SD)	3.200 (2.683)	12.865 (9.681)	11.516 (9.626)		**0.021**
Extrathyroid extension No, *N* (%) Yes, *N* (%)	6 (100.0%) 0 (0.0%)	34 (91.9%) 3 (8.1%)	40 (93.0%) 3 (7.0%)		0.470
Vascular invasion No, *N* (%) Yes, *N* (%)	6 (100.0%) 0 (0.0%)	21 (56.8%) 16 (43.2%)	27 (62.8%) 16 (37.2%)		**0.042**
Perineural invasion No, *N* (%) Yes, *N* (%)	6 (100.0%) 0 (0.0%)	35 (94.6%) 2 (5.4%)	41 (95.3%) 2 (4.7%)		0.560
Encapsulated neoplasm No, *N* (%) Yes, *N* (%)	6 (100.0%) 0 (0.0%)	35 (94.6%) 2 (5.4%)	41 (95.3%) 2 (4.7%)		0.560
Capsular infiltration No, *N* (%) Yes, *N* (%)	6 (100.0%) 0 (0.0%)	35 (94.6%) 2 (5.4%)	41 (95.3%) 2 (4.7%)		0.560
Surgical margins Negative, *N* (%) Positive, *N* (%)	6 (100.0%) 0 (0.0%)	35 (94.6%) 2 (5.4%)	41 (95.3%) 2 (4.7%)		0.560
Multifocality No, *N* (%) Yes, *N* (%)	5 (83.3%) 1 (16.7%)	17 (45.9%) 20 (54.1%)	22 (51.2%) 21 (48.8%)		0.089
Stage at diagnosis, *N* (%) I II III IVa Ivb IVc	6 (100.0%) 0 (0.0%) 0 (0.0%) 0 (0.0%) 0 (0.0%) 0 (0.0%)	20 (54.1%) 2 (5.4%) 3 (8.1%) 10 (27.0%) 0 (0.0%) 2 (5.4%)	26 (60.5%) 2 (4.7%) 3 (7.0%) 10 (23.3%) 0 (0.0%) 2 (4.7%)	0.336	
DFS, months Mean (SD)	66.167 (70.130)	46.088 (62.210)	49.100 (62.912)	0.478	
PFS, months Mean (SD)	0.0 (0.0)	48.667 (66.154)	48.667 (66.154)		
Pre-surgery calcitonin, pg/ml Mean (SD)	65.750 (66.965)	2008.354 (7,017.423)	1737.293 (6,532.515)	0.506	
Post-surgery calcitonin, pg/ml Mean (SD)	2.175 (1.278)	246.514 (770.471)	212.420 (718.444)	0.446	
Last follow-up calcitonin, pg/ml Mean (SD)	1.758 (1.533)	215.041 (651.506)	185.280 (607.795)	0.432	
Last follow-up CEA, ng/ml Mean (SD)	1.480 (1.265)	43.328 (150.068)	37.489 (139.710)	0.503	
Last follow-up status, *N* (%) Cured Not cured	6 (100.0%) 0 (0.0%)	20 (54.1%) 17 (45.9%)	26 (60.5%) 17 (39.5%)	**0.033**	

Compared with INSM1-negative tumours, INSM1-positive tumours displayed:

- Larger size (12.9 ± 9.7 mm vs 3.2 ± 2.7 mm, *p* = 0.021).
- Vascular invasion in 43.2% vs 0% (*p* = 0.042).

Stage distribution, DFS, PFS, calcitonin/CEA values, and double tumours frequency did not differ significantly (all *p* > 0.33). Cure at last follow-up occurred in 6/6 INSM1-negative (100%) versus 20/37 INSM1-positive patients (54.1%; *p* = 0.033).

SSTR2A correlations (Table 3, Supplementary Materials Figures SC and SD).

**Table 3:** SSTR2A

	SSTR2A negative		SSTR2A positive	Total		Test (*p*-value)	Odds ratio (OR) 95% CI
Number of patients, *N* (%)	37 (86.0%)		6 (14.0%)	43 (100.0%)			
Type Only medullary cancer, *N* (%) Double tumours *N* (%)	28 (75.7%) 9 (24.3%)		2 (33.3%) 4 (66.7%)	30 (69.8%) 13 (30.2%)		**0.036**	6.222222 0.9724119—39.81445
Primary tumour size, mm Mean (SD)	12.208 (10.153)		7.250 (3.283)	11.516 (9.626)		0.247	
Extrathyroid extension No,* N* (%) Yes, *N* (%)	34 (91.9%) 3 (8.1%)		6 (100.0%) 0 (0.0%)	40 (93.0%) 3 (7.0%)		0.470	
Vascular invasion No, *N* (%) Yes, *N* (%)	23 (62.2%) 14 (37.8%)		4 (66.7%) 2 (33.3%)	27 (62.8%) 16 (37.2%)		0.832	
Perineural invasion No, *N* (%) Yes, *N* (%)	35 (94.6%) 2 (5.4%)		6 (100.0%) 0 (0.0%)	41 (95.3%) 2 (4.7%)		0.560	
Encapsulated neoplasm No,* N* (%) Yes, *N* (%)	35 (94.6%) 2 (5.4%)		6 (100.0%) 0 (0.0%)	41 (95.3%) 2 (4.7%)		0.560	
Capsular infiltration No, *N* (%) Yes, *N* (%)	35 (94.6%) 2 (5.4%)		6 (100.0%) 0 (0.0%)	41 (95.3%) 2 (4.7%)		0.560	
Surgical margins Negative, *N* (%) Positive, *N* (%)	35 (94.6%) 2 (5.4%)		6 (100.0%) 0 (0.0%)	41 (95.3%) 2 (4.7%)		0.560	
Multifocality No, *N* (%) Yes, *N* (%)	18 (48.6%) 19 (51.4%)		4 (66.7%) 2 (33.3%)	22 (51.2%) 21 (48.8%)		0.413	
Stage at diagnosis, *N* (%) I II III IVa IVb IVc	21 56.8%) 2 (5.4%) 3 (8.1%) 9 (24.3%) 0 (0.0%) 2 (5.4%)	5 (83.3%) 0 (0.0%) 0 (0.0%) 1 (16.7%) 0 (0.0%) 0 (0.0%)		26 (60.5%) 2 (4.7%) 3 (7.0%) 10 (23.3%) 0 (0.0%) 2 (4.7%)	TEST (*p*-value) 0.760		
DFS, months Mean (SD)	53.882 (67.113)	22.000 (10.354)		49.100 (62.912)	0.258		
PFS, months Mean (SD)	48.667 (66.154)	0.0 (0.0)		48.667 (66.154)			
Pre-surgery calcitonin, pg/ml Mean (SD)	2005.838 (7018.033)	81.267 (122.138)		1737.293 (6532.515)	0.510		
Post-surgery calcitonin, pg/ml Mean (SD)	246.122 (770.595)	4.592 (5.810)		212.420 (718.444)	0.452		
Last follow-up calcitonin, pg/ml Mean (SD)	214.986 (651.523)	2.092 (3.393)		185.280 (607.795)	0.433		
Last follow-up CEA, ng/ml Mean (SD)	43.384 (150.053)	1.137 (0.877)		37.489 (139.710)	0.499		
Last follow-up status, *N* (%) Cured Not cured	20(54.1%) 17(45.9%)	6 (100.0%) 0 (0.0%)		26 (60.5%) 17 (39.5%)	**0.033**		

SSTR2A positivity was associated with:

- Double tumours in 4/6 cases (66.7%) vs 9/37 SSTR2A-negative (24.3%; *p* = 0.036; OR 6.22, 95% CI 0.97–39.81)
- Biochemical cure in *6/6 patients (100%)* vs 20/37 (54.1%; *p* = 0.033)

No significant differences emerged for tumour size, vascular invasion, extrathyroid extension, DFS, PFS, or serum calcitonin/CEA (all *p* > 0.05), as well as desmoplasia in MTC and the genetic profile of the tumours.

### Comorbidities and outcome (supplementary materials Table S5)

Associations between comorbidities and cure status are shown in Supplementary Materials Table S5. None of the conditions analysed (hypertension, diabetes, dyslipidaemia, osteoporosis, pheochromocytoma, hyperparathyroidism, other malignancies) correlated significantly with outcome (all *p* > 0.13).

### Double tumours (supplementary materials Table S6 and supplementary materials Figure SC)

Double tumours cases (13/43; 30.2%) are compared with cases defined by only medullary carcinomas in Supplementary Materials Table S6 and depicted in Supplementary Materials Figure SC. The double tumours cohort showed a higher prevalence of SSTR2A positivity (30.8% vs 6.7%, *p* = 0.036), whereas Ki-67 category and INSM1 expression were comparable between groups. Stage at diagnosis, primary tumour size, vascular/perineural or capsular invasion, and margin status likewise did not differ significantly. Double tumours cases tended to display lower pre- and post-operative calcitonin levels and no metastases at last follow-up, but these trends did not reach statistical significance.

## Discussion

The current study analyses a retrospective series of MTC which were studied with an immunopanel in order to discuss the possible diagnostic and prognostic implication. MTC accounts at around 2% of all thyroid malignancies, representing the third most frequent type of thyroid malignancy after papillary and follicular carcinoma; it occurs as 70–75% sporadic whilst 20–25% are hereditary cases [5]. The 5th edition of the Endocrine and Neuroendocrine WHO 2022 book introduced the subclassification as low-grade and high-grade MTC based on the detection of the following parameters: (1) Ki-67 ≥ 5%; (2) > 5 mitoses per 2 mm^2^, necrotic component; this new subclassification recognizes approximately 25% of MTC as high-grade subtype [14]. A definition of parameters able to correctly stratify MTC is extremely crucial and relevant for the follow-up and management. In this single-centre series, a 3% Ki-67 threshold already delineated medullary thyroid carcinomas (MTCs) that were larger and much more likely to exhibit vascular invasion than their low-proliferation counterparts. The observation echoes the low-range cut-off identified by Fuchs et al. in 140 cases [5] and remains broadly compatible with the 5% boundary adopted by the International Medullary Thyroid Carcinoma Grading System (IMTCGS) [7]. The reason of studying this 3% value is also due to the evidence, whilst we analysed the series, that many cases had a Ki-67 lower than 5% but not 0%, so we arbitrarily chose 3% and compare with the standard 5%. Because a Ki-67 index ≤ 5% is encountered in roughly two-thirds of routine pathology reports, confirmation of its prognostic meaning at the 3% level would have immediate clinical value: tumours crossing this low bar could be flagged for closer surveillance even when classified as early stage. Initially, we applied the WHO 2022 classification 5% cut-off to dichotomise tumours into low- and high-grade categories; however, only 2 of 43 cases (4.7%) fell into the ≥ 5% group, and no clinicopathological or outcome variable reached statistical significance (all *p* > 0.05) [14]. Because of this limited discriminatory power, we re-set the threshold to 0–3% versus > 3%. Our purpose was to look at those cases with a Ki-67 lower than 5% but not close to 0 and verify if there is any correlation with clinic-pathological findings. Although not perfectly superimposable on the < 3%/≥ 3% scheme of Fuchs et al., the new split retains the same rationale of interrogating the lower proliferative range [6]. Under this definition, tumours with Ki-67 > 3% were significantly larger and exhibited an almost threefold increase in vascular invasion, while all other parameters remained unchanged. These findings suggest that a 3% Ki-67 threshold may provide more informative prognostic stratification, particularly in cohorts in which most MTCs display indices between 1 and 5%. In such settings, surpassing even the 3% mark could serve as an early warning of more aggressive biological behaviour, warranting closer follow-up—yet still aligning with the IMTCGS concept of high-grade at ≥ 5%. Multicentre studies with larger sample sizes are required to validate this intermediate cut-off. Beyond proliferation, *INSM1 positivity* tracked the same adverse histological features and was associated with about half the biochemical-cure rate recorded in INSM1-negative tumours. To date, INSM1 has been framed almost exclusively as a diagnostic marker with good sensitivity and specificity for MTC [9]. INSM1 is a cytoplasmic neuroendocrine marker of tumour cells expressed in pancreas, brain tumours, pheochromocytomas, medullary thyroid carcinomas, insulinomas, and pituitary and small-cell lung carcinomas [27]. However, experimental data in other neuro-endocrine malignancies—such as nasopharyngeal carcinoma, where high INSM1 predicts radio-resistance and worse outcome [10]—support a broader role in tumour biology. Indeed recently, INSM1 has emerged as a promising marker of tumour aggressiveness across several neuroendocrine neoplasms, such as olfactory neuroblastoma and high-grade neuroendocrine breast tumours where high INSM1 expression was significantly associated with higher histologic grade and poorer prognosis [28, 29].

Our findings extend this possibility to thyroid C-cell neoplasia and suggest that INSM1 could complement Ki-67 when identifying high-risk disease immediately after surgery.

Membranous SSTR2A expression identified a small subset of tumours in our cohort, all of which achieved biochemical cure. MTC tissue has been shown to express somatostatin receptors (SSTRs) [30], and both in vitro and in vivo studies have highlighted the potential role of SSAs in MTC management, either as monotherapy or in combination with other drugs. Despite conflicting findings from Herac et al. [31], more recent work by de Vries et al. suggested that SSTR2A expression correlates with longer overall survival in MTC patients, particularly in stage IV, indicating its potential as a prognostic marker [8].

Interestingly, the cases with SSTR2A positivity were disproportionately represented among patients with double tumours, defined as the synchronous presence of two distinct histological subtypes, a medullary thyroid carcinoma (MTC) and a non-medullary component, most frequently papillary thyroid carcinoma (PTC), occurring in separate areas of the gland. In our series, the prevalence of such double tumours reached 30%, which exceeds the ~ 19–20% reported in studies focused on MTC patients [32]. This discrepancy likely reflects referral bias, as our centre serves as a tertiary referral institution for advanced or complex MTC cases and may also be influenced by the limited sample size. In our single-centre cohort, 13 out of 43 patients (30.2%) presented with a double tumour, defined as the synchronous occurrence of two distinct neoplastic components: medullary thyroid carcinoma (MTC) and a differentiated non-medullary carcinoma, predominantly papillary thyroid carcinoma (PTC), in anatomically separated regions of the thyroid gland. Despite minor differences in incidence, the clinicopathological behaviour of our double tumour cases closely mirrored that of pure medullary thyroid carcinoma (MTC). Tumour size, as well as rates of vascular, perineural, and capsular invasion and proliferative indices, showed no significant differences between double tumours and solitary MTC. This observation aligns with the Italian multicentre study by Appetecchia et al. (2019) and recent data from the National Cancer Database (NCDB) (2023), which indicate that outcomes in synchronous MTC/PTC are primarily driven by the medullary component [33, 34]. We also found that pre- and postoperative calcitonin concentrations were generally lower in the double-tumour subgroup, likely reflecting a reduced medullary tumour burden, a trend evident in Chinese series, particularly in cases of micro-MTC < 1 cm [35, 36]. The strengths of the study include uniform surgical treatment, central pathology review, and long medium follow-up. Limitations of the present work include its retrospective nature, the relatively limited sample size, and the low number of clinical events, which restricted the possibility of performing robust multivariable survival analyses. Furthermore, as some variables are likely to be collinear—for instance, tumour size and Ki-67 index—it remains to be clarified whether the markers investigated provide independent prognostic information or rather reflect tumour aggressiveness already appreciable through standard histological assessment. Future multicentre studies should address these aspects, with the dual aim of validating the most appropriate Ki-67 threshold, assessing inter-observer reproducibility of INSM1 scoring, and further defining the role of SSTR2A immunohistochemistry as a consistent and reliable prognostic marker.

In conclusion, Ki-67 > 3%, INSM1 positivity, and absence of membranous SSTR2A identify an MTC subgroup characterised by larger primary tumours, vascular invasion, and reduced biochemical/structural cure rates, whereas SSTR2A-positive tumours—often double tumours—showed uniformly favourable early outcomes in our series. Immunoscoring of this three-marker panel may refine postoperative risk stratification and personalise follow-up, but prospective multi-centre validation is required before routine clinical adoption.

## Supplementary Information

Below is the link to the electronic supplementary material.

ESM 1 (DOCX 709 KB)
