# Supplementary material for: Immunohistochemical expression of Ki-67, INSM1, and SSTR2A in medullary thyroid carcinoma: correlation with tumour size, vascular invasion, and biochemical outcome
Source: Virchows Arch. 2025 Oct 25;489(2):427–39. doi: 10.1007/s00428-025-04287-z (PMC13477441; doi:10.1007/s00428-025-04287-z)
Supplement: Supplementary file 1 [file 428_2025_4287_MOESM1_ESM.pdf]

SUPPLEMENTARY MATERIALS

Table S1 (PATIENTS' CHARACTERISTICS 1)

Variable	Category	Freq. (n)	Percent (%)	Cumulative (%)
Sex	Male	17	39.53	39.53
	Female	26	60.47	100.0
Thyroid surgery	Yes	43	100.0	100.0
Somatic RET mutation (n=9)	No	8	88.89	88.89
	Yes	1	11.11	100.0
MEN2A (n=11)	No	8	72.73	72.73
	Yes	3	27.27	100.0
MEN2B (n=11)	No	10	90.91	90.91
	Yes	1	9.09	100.0
FDG-PET	Negative/not performed	40	93.02	93.02
	Positive	3	6.98	100.0
Gallium68-PET	Negative/not performed	43	100.0	100.0
DOPA-PET	Negative/not performed	35	81.4	81.4
	Positive	8	18.6	100.0
Last follow-up Status	Cured	26	60.47	60.47
	Not cured	17	39.53	100.0
Metastasis at Last Follow-up	No	36	83.72	83.72
	Yes	7	16.28	100.0
Systemic Therapy	No	39	90.7	90.7
	Yes	4	9.3	100.0

Table S2 (PATIENTS' CHARACTERISTICS 2)

Variable	Category	Freq. (n)	Percent (%)	Cumulative (%)
Tumor Type	Only medullary cancer	30	69.77	69.77
	Double tumors	13	30.23	100.0
Stage at diagnosis	I	26	60.47	60.47
	II	2	4.65	65.12
	III	3	6.98	72.09
	IVA	10	23.26	95.35
	IVC	2	4.65	100.0
T stage	T1a	25	58.14	58.14
	T1b	10	23.26	81.4
	T2	3	6.98	88.37
	T3a	3	6.98	95.35
	T4a	2	4.65	100.0
N stage	N0	29	67.44	67.44
	N1a	4	9.3	76.74
	N1b	10	23.26	100.0
M stage	M0	41	95.35	95.35
	M1	2	4.65	100.0
Extrathyroid extension	No	40	93.02	93.02
	Yes	3	6.98	100.0
Vascular invasion	No	27	62.79	62.79
	Yes	16	37.21	100.0
Perineural invasion	No	41	95.35	95.35
	Yes	2	4.65	100.0
Encapsulated neoplasm	No	41	95.35	95.35
	Yes	2	4.65	100.0

Capsular infiltration	No	41	95.35	95.35
	Yes	2	4.65	100.0
Surgical margins	Negative	41	95.35	95.35
	Positive	2	4.65	100.0
Multifocality	No	22	51.16	51.16
	Yes	21	48.84	100.0

Table S3 (PATIENTS' CHARACTERISTICS 3)

Variable	Mean	Std. dev.	Variance	Skewness	Kurtosis
Age at diagnosis (years)	54.34884	15.29221	233.8516	-0.0308773	2.028669
Primary tumor size (mm)	11.51628	9.62645	92.66854	2.024632	7.578509
Pre-surgery Calcitonin (pg/ml)	1737.293	6532.515	42700000.0	5.70827	35.48947
Post-surgery Calcitonin (pg/ml)	212.4198	718.4437	516161.3	3.95343	17.40085
Last follow-up Calcitonin (pg/ml)	185.2802	607.7949	369414.6	4.105559	19.13593
Last follow-up CEA (ng/ml)	37.48907	139.7096	19518.76	4.833083	26.55489
Disease-free survival (months)	49.1	62.91174	3957.887	2.095722	6.106124

Progression-free survival (months) (n=3)	48.66667	66.15386	4376.333	0.7025652	1.5
Mean Follow-up duration (months)	52.46512	65.35629	4271.445	1.973686	5.509953

Table S4 (PATIENTS' CHARACTERISTICS 4)

Variable	Category	Freq. (n)	Percent (%)	Cumulative (%)
Hypertension (n=42)	No	27	64.29	64.29
	Yes	15	35.71	100.0
Diabetes (n=42)	No	37	88.1	88.1
	Yes	5	11.9	100.0
Osteopenia/Osteoporosis (n=42)	No	36	85.71	85.71
	Yes	6	14.29	100.0
Other Non-Thyroid Neoplasms (n=42)	No	35	83.33	83.33
	Yes	7	16.67	100.0
Pheochromocytoma (n=42)	No	38	90.48	90.48
	Yes	4	9.52	100.0
Hyperparathyroidism (n=42)	No	38	90.48	90.48
	Yes	4	9.52	100.0
Dyslipidemia	No	29	67.44	67.44
	Yes	14	32.56	100.0

Table S5 (COMORBIDITIES Vs OUTCOME)

	Cured	Not cured	TOTAL	TEST (p-value)
Pheochromocytoma N (%)<br>No<br>Yes	 24 (96.0%)<br>1 (4.0%)	 14 (82.4%)<br>3 (17.6%)	 38 (90.5%)<br>4 (9.5%)	 0.139
Hyperparathyroidism N (%)<br>No<br>Yes	 24 (96.0%)<br>1 (4.0%)	 14 (82.4%)<br>3 (17.6%)	 38 (90.5%)<br>4 (9.5%)	 0.139
Dyslipidemia N (%)<br>No<br>Yes	 16 (61.5%)<br>10 (38.5%)	 13 (76.5%)<br>4 (23.5%)	 29 (67.4%)<br>14 (32.6%)	 0.307
Hypertension N (%)<br>No<br>Yes	 17 (68.0%)<br>8 (32.0%)	 10 (58.8%)<br>7 (41.2%)	 27 (64.3%)<br>15 (35.7%)	 0.542
Diabetes N (%)<br>No<br>Yes	 21 (84.0%)<br>4 (16.0%)	 16 (94.1%)<br>1 (5.9%)	 37 (88.1%)<br>5 (11.9%)	 0.320
Osteopenia/Osteoporosis N (%)<br>No<br>Yes	 23 (92.0%)<br>2 (8.0%)	 13 (76.5%)<br>4 (23.5%)	 36 (85.7%)<br>6 (14.3%)	 0.158
Other Non-Thyroid Neoplasms N (%)<br>No<br>Yes	 21 (84.0%)<br>4 (16.0%)	 14 (82.4%)<br>3 (17.6%)	 35 (83.3%)<br>7 (16.7%)	 0.888

Table S6 (TUMOR TYPE)

	PURE MTC	DOUBLE TUMORS	TOTAL	TEST (p-value)
Number of patients<br>N (%)	30 (69.8 %)	13 (30.2 %)	43 (100 %)	
Sex N (%)<br>M<br>F	 12 (40.0 %);<br>18 (60.0 %)	 5 (38.5 %);<br>8 (61.5 %)	 17 (39.5 %);<br>26 (60.5 %)	0.925
Age at diagnosis (yrs)<br>Mean (SD)	53.033<br>(15.446)	57.385<br>(15.086)	54.349<br>(15.292)	0.398
Stage at Diagnosis N (%)<br>I<br>II<br>III<br>IVa<br>IVb<br>IVc	 18 (60.0 %);<br>1 (3.3 %);<br>2 (6.7 %);<br>7 (23.3 %);<br>0 (0.0%);<br>2 (6.7 %)	 8 (61.5 %);<br>1 (7.7 %);<br>1 (7.7 %);<br>3 (23.1 %);<br>0 (0.0%);<br>0 (0 %)	 26 (60.5 %);<br>2 (4.7 %);<br>3 (7.0 %);<br>10 (23.3 %);<br>0 (0.0%);<br>2 (4.7 %)	0.869
Primary-tumour size (mm)<br>Mean (SD)	11.690<br>(10.717)	11.115 (6.820)	11.516 (9.626)	0.860
Extrathyroid Extension N (%)<br>No<br>Yes	 28 (93.3 %);<br>2 (6.7 %)	 12 (92.3 %);<br>1 (7.7 %)	 40 (93.0 %);<br>3 (7.0 %)	0.903
Vascular Invasion N (%)<br>No<br>Yes	 17 (56.7 %);<br>13 (43.3 %)	 10 (76.9 %);<br>3 (23.1 %)	 27 (62.8 %);<br>16 (37.2 %)	0.207
Perineural Invasion N (%)<br>No<br>Yes	 28 (93.3 %);<br>2 (6.7 %)	 13 (100 %);<br>0 (0 %)	 41 (95.3 %);<br>2 (4.7 %)	0.340

Encapsulated neoplasm N (%)<br>No<br>Yes	 28 (93.3 %);<br>2 (6.7 %)	 13 (100 %);<br>0 (0 %)	 41 (95.3 %);<br>2 (4.7 %)	 0.340
Capsular infiltration N (%)<br>No<br>Yes	 28 (93.3 %);<br>2 (6.7 %)	 13 (100 %);<br>0 (0 %)	 41 (95.3 %);<br>2 (4.7 %)	 0.340
Positive surgical margins N (%)<br>No<br>Yes	 28 (93.3 %);<br>2 (6.7 %)	 13 (100 %);<br>0 (0 %)	 41 (95.3 %);<br>2 (4.7 %)	 0.340
Multifocality<br>N (%)<br>No<br>Yes	 20 (66.7 %);<br>10 (33.3 %)	 2 (15.4 %);<br>11 (84.6 %)	 22 (51.2 %);<br>21 (48.8 %)	 **0.002**
Ki-67 index<br>N (%)<br>0-3%<br>>3%	 26 (86.7 %);<br>4 (13.3 %);	 12 (92.3 %);<br>1 (7.7 %);	 38 (88.4 %);<br>5 (11.6%);	 0.772
SSTR2 expression N (%)<br>Negative<br>Positive	 28 (93.3 %);<br>2 (6.7 %)	 9 (69.2 %);<br>4 (30.8 %)	 37 (86.0 %);<br>6 (14.0 %)	 **0.036**
INSM1 expression N (%)<br>Negative<br>Positive	 5 (16.7 %);<br>25 (83.3 %)	 1 (7.7 %);<br>12 (92.3 %)	 6 (14.0 %);<br>37 (86.0 %)	 0.435
Pre-surgery calcitonin (pg/ml)<br>Mean (SD)	 2 358.237<br>(7 771.791)	 304.346<br>(448.672)	 1 737.293<br>(6 532.515)	 0.350
Post-surgery calcitonin (pg/ml)<br>Mean (SD)	296.450<br>(850.384)	18.504<br>(23.553)	212.420<br>(718.444)	0.249
Last FU calcitonin (pg/ml)<br>Mean (SD)	260.533<br>(717.964)	11.619<br>(19.907)	185.280<br>(607.795)	0.222
Last FU CEA (ng/ml)<br>Mean (SD)	52.700<br>(165.754)	2.386 (2.442)	37.489<br>(139.710)	0.283

Disease-free survival (mo) Mean (SD)	56.037 (68.437)	34.692 (48.806)	49.100 (62.912)	0.321
Not cured at last FU N (%)	12 (40.0 %)	3 (23.1 %)	15 (34.9 %)	0.285
Metastasis at last FU N (%)	7 (23.3 %)	0 (0 %)	7 (16.3 %)	0.057

Figure SA

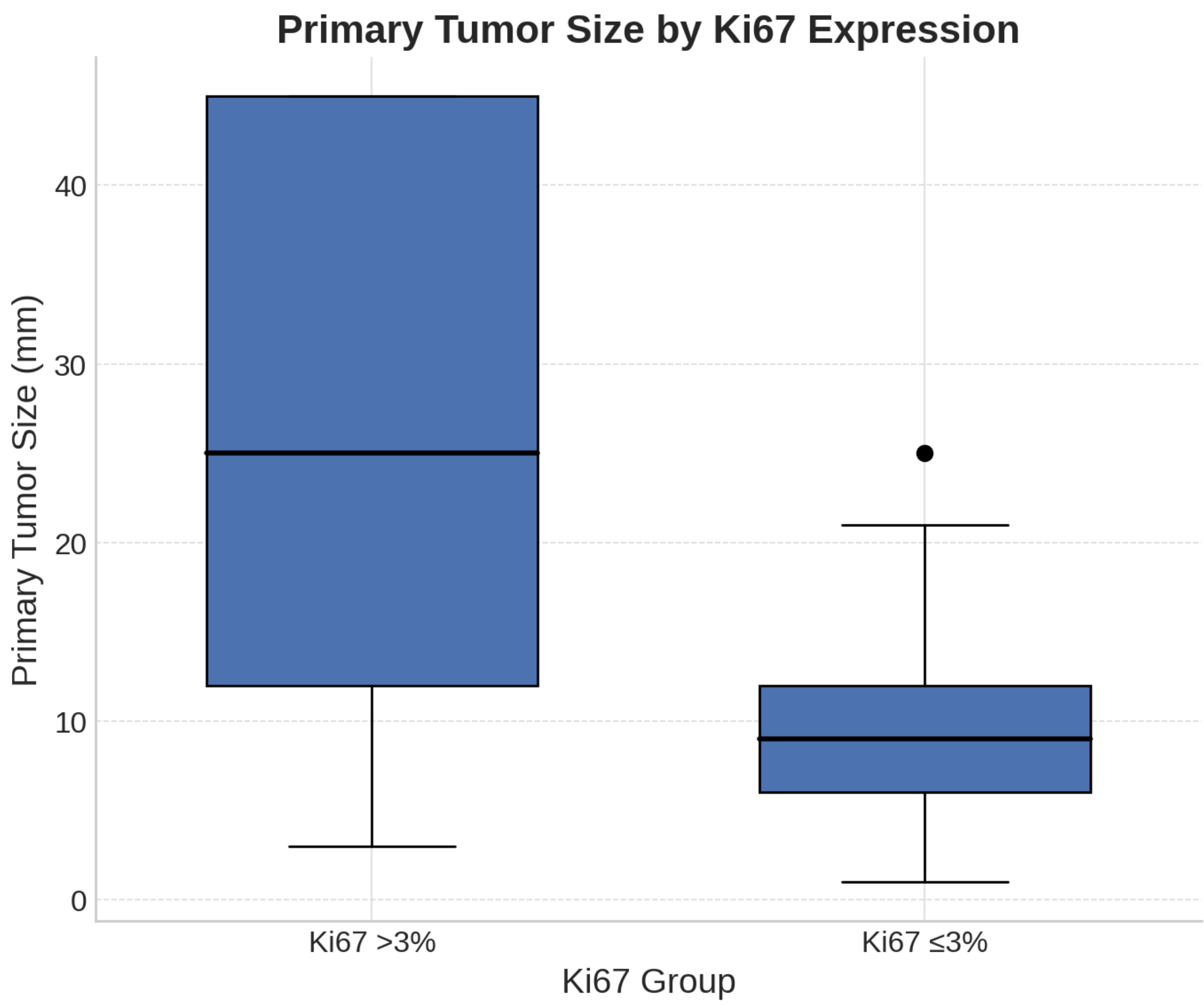

Figure SB

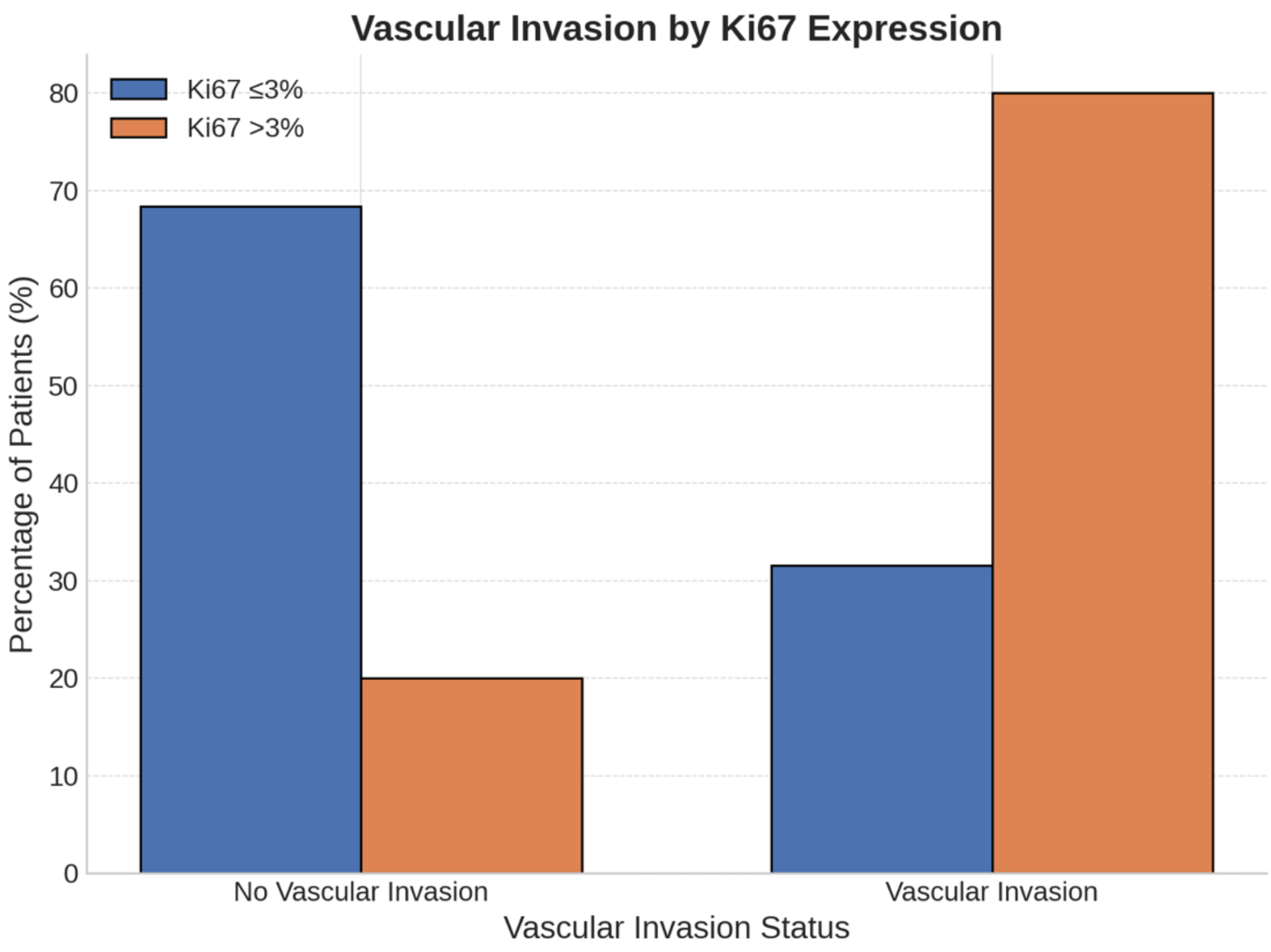

Figure SC

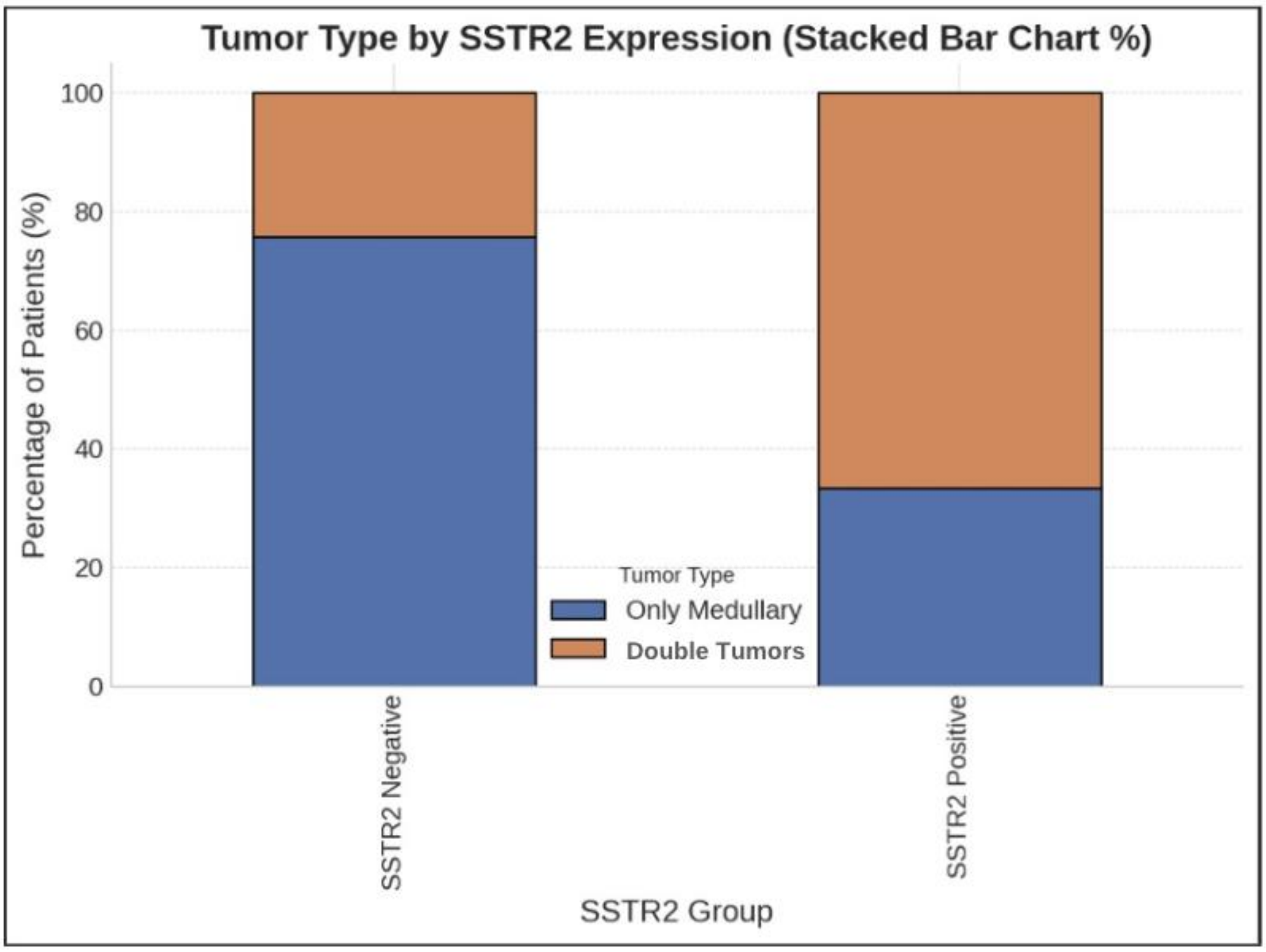

Figure SD

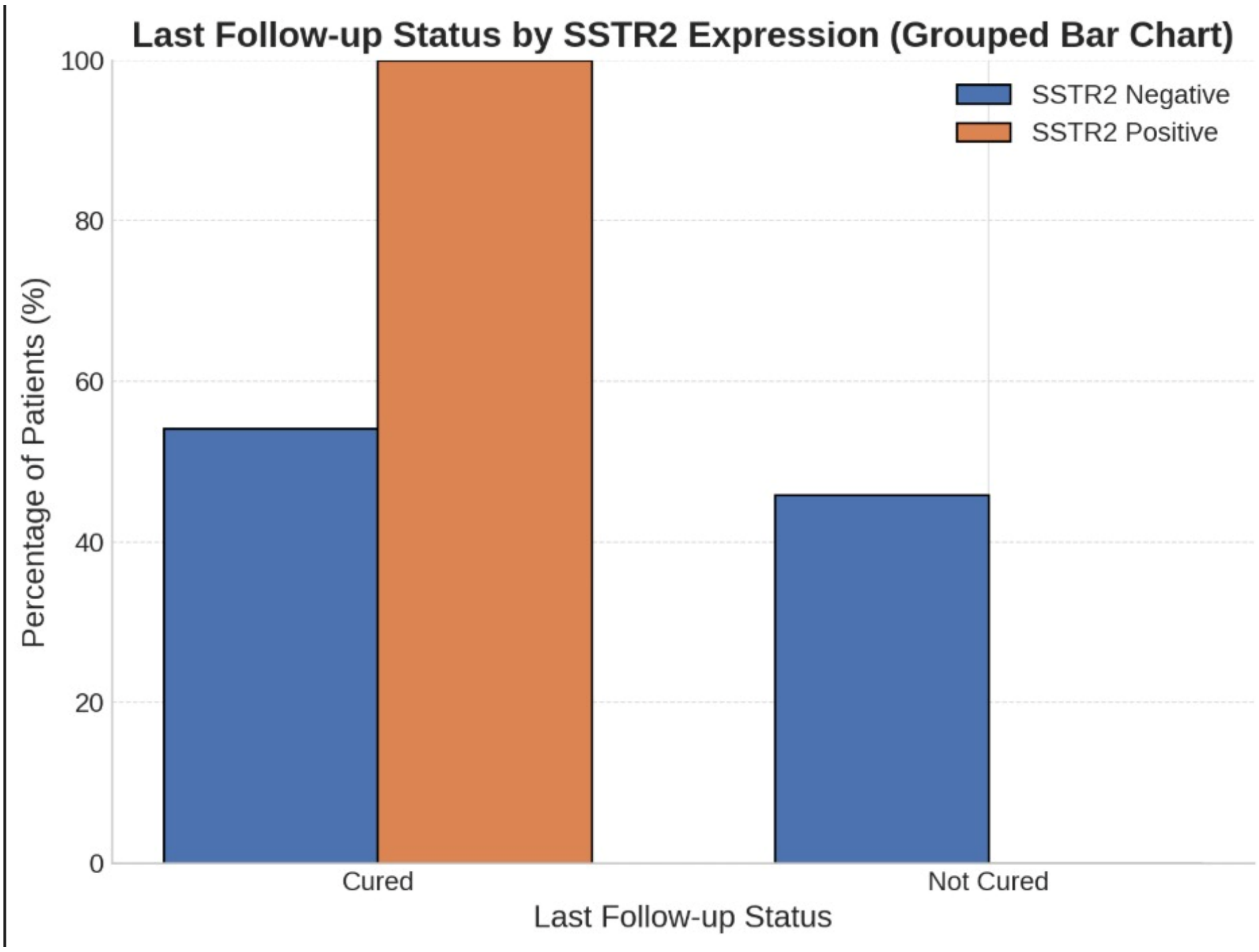

Figure SE

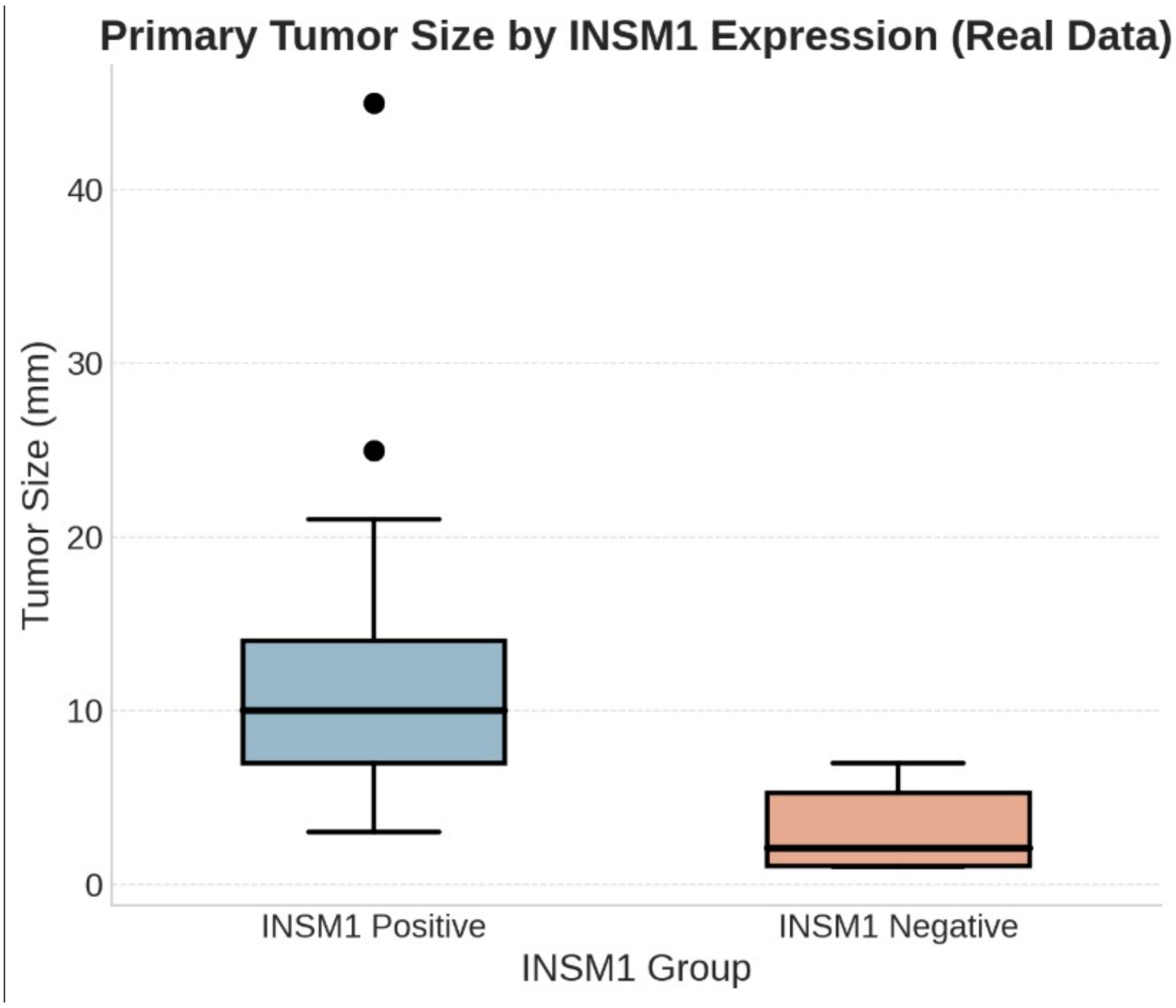

Figure SF

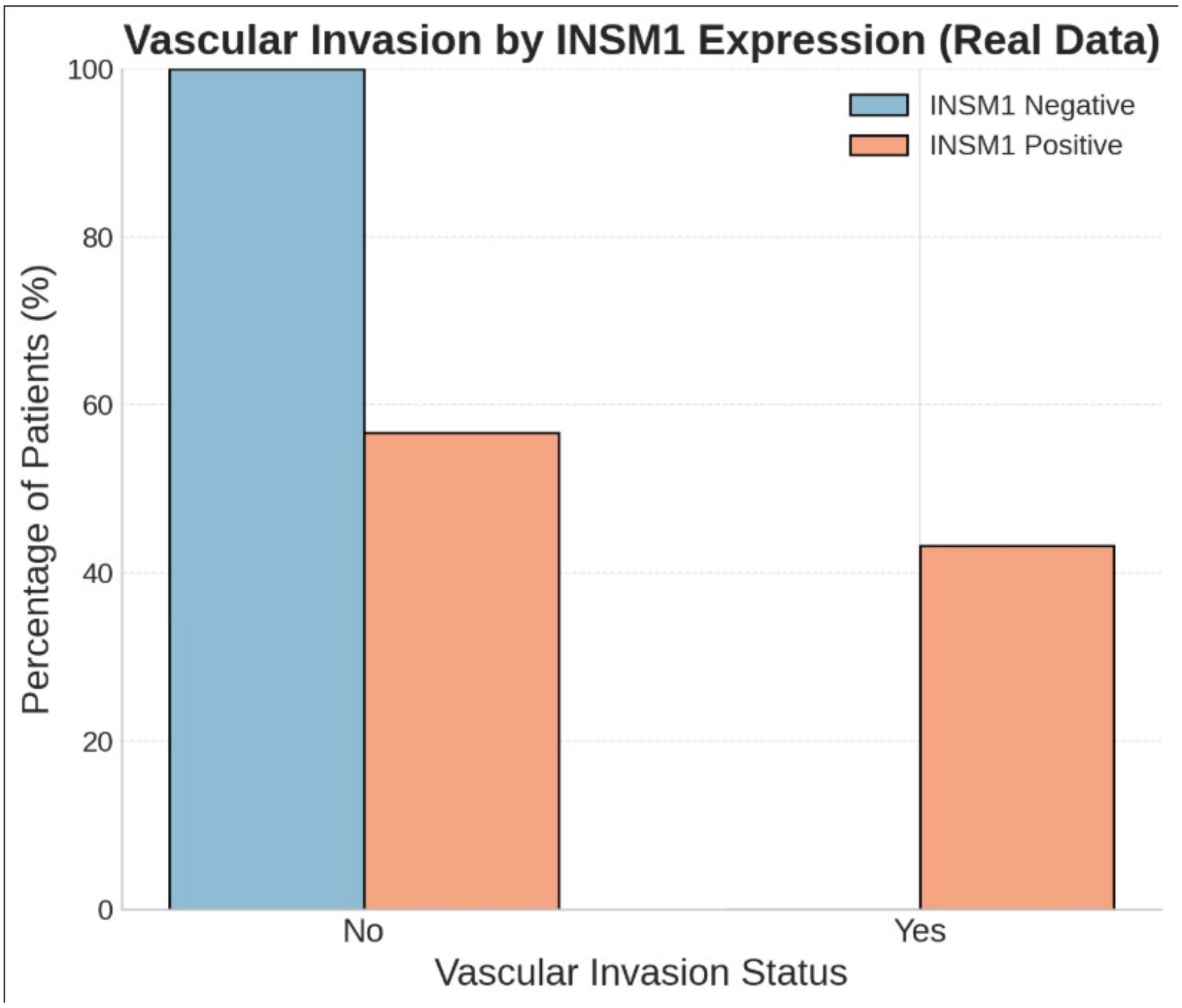
Vascular Invasion by INSM1 Expression (Real Data)
INSM1 Negative
INSM1 Positive
Percentage of Patients (%)
100
80
60
40
20
0
No
Yes
Vascular Invasion Status

Figure SG

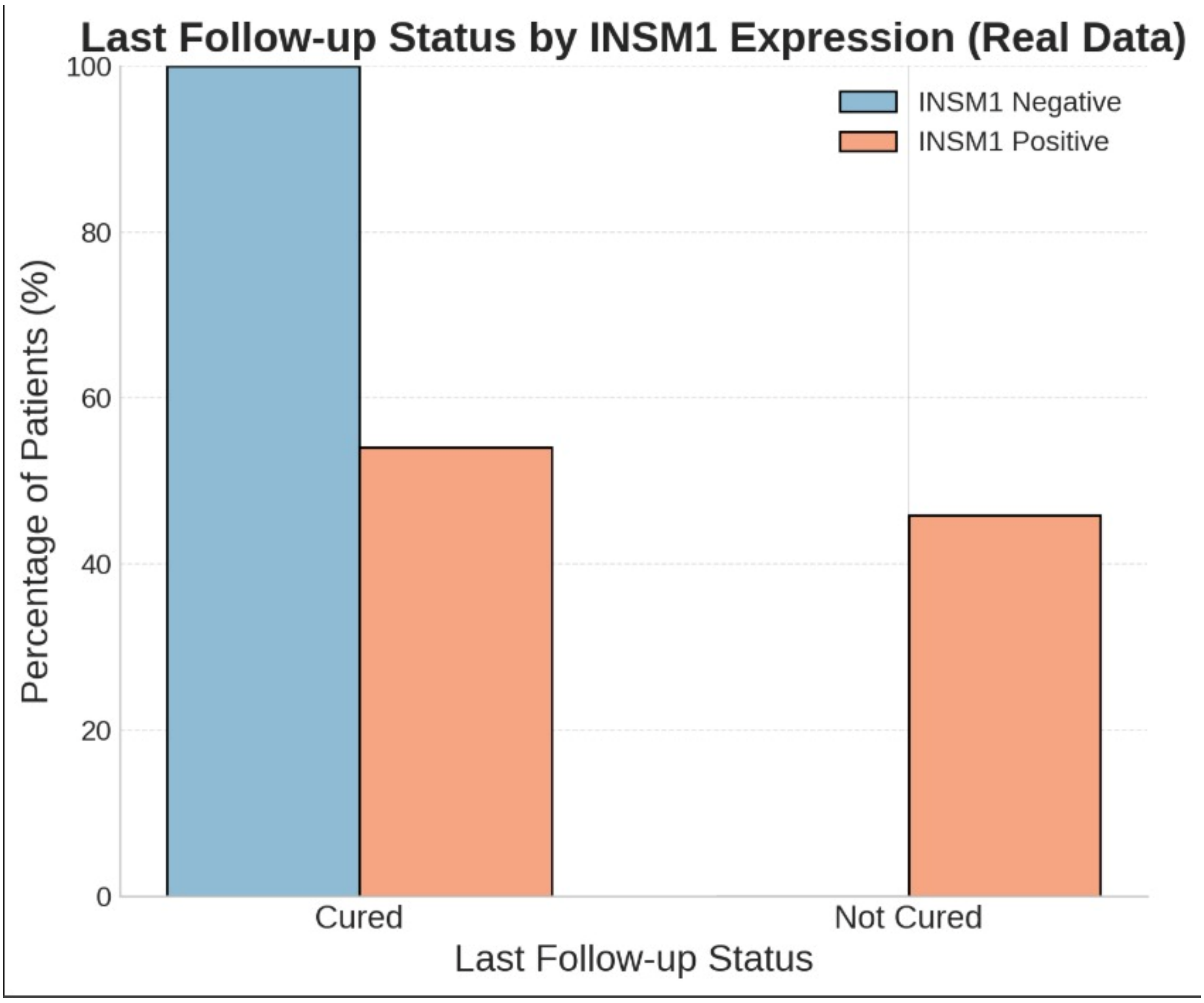
Last Follow-up Status by INSM1 Expression (Real Data)
INSM1 Negative
INSM1 Positive
Percentage of Patients (%)
100
80
60
40
20
0
Cured
Not Cured
Last Follow-up Status
